# A second polymorph of aqua­{4-chloro-2-[(pyridin-2-ylmeth­yl)imino­meth­yl]­phenolato}copper(II) nitrate mono­hydrate

**DOI:** 10.1107/S1600536812004564

**Published:** 2012-02-10

**Authors:** Jing Yu

**Affiliations:** a College of Biological and Chemical Sciences Engineering, Jiaxing University, Jiaxing Zhejiang 314001, People’s Republic of China

## Abstract

The title complex, [Cu(C_13_H_10_ClN_2_O)(H_2_O)]NO_3_·H_2_O, was obtained by the reaction of 5-chloro­salicyl­aldehyde, 2-(amino­meth­yl)pyridine and copper nitrate in methanol. The first reported polymorph of this complex was triclinic [Liang *et al.* (2010 ▶). *Acta Cryst.* E**66**, m40]. The present polymorph crystallized in the monoclinic space group *P*2_1_/*c*. The Cu^II^ ion is in a square planar environment and is coordinated by one phenolate O, one imine N and one pyridine N atom of the tridentate Schiff base ligand and by one water O atom. In the crystal, mol­ecules are linked through inter­molecular O—H⋯O hydrogen bonds to form chains along the *a* axis.

## Related literature

For the structures and properties of Schiff base copper(II) complexes, see: Patel *et al.* (2011 ▶); Creaven *et al.* (2010 ▶); Osowole *et al.* (2008 ▶). For the complex with triclinic space group *P* 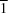, see: Liang *et al.* (2010 ▶).
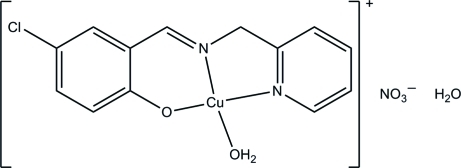


## Experimental

### 

#### Crystal data

[Cu(C_13_H_10_ClN_2_O)(H_2_O)]NO_3_·H_2_O
*M* *_r_* = 407.26
Monoclinic, 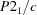

*a* = 7.840 (2) Å
*b* = 8.815 (3) Å
*c* = 23.079 (3) Å
β = 99.680 (2)°
*V* = 1572.4 (7) Å^3^
*Z* = 4
Mo *K*α radiation
μ = 1.60 mm^−1^
*T* = 298 K
0.22 × 0.20 × 0.19 mm

#### Data collection

Bruker SMART 1K CCD area-detector diffractometer
Absorption correction: multi-scan (*SADABS*; Sheldrick, 2004 ▶) *T*_min_ = 0.720, *T*_max_ = 0.752
12290 measured reflections
3410 independent reflections
2647 reflections with *I* > 2σ(*I*)
*R*_int_ = 0.046

#### Refinement

*R*[*F* ^2^ > 2σ(*F* ^2^)] = 0.037
*wR*(*F* ^2^) = 0.089
*S* = 1.06
3410 reflections
233 parameters
3 restraints
H atoms treated by a mixture of independent and constrained refinement
Δρ_max_ = 0.39 e Å^−3^
Δρ_min_ = −0.48 e Å^−3^

### 

Data collection: *SMART* (Bruker, 2001 ▶); cell refinement: *SAINT* (Bruker, 2001 ▶); data reduction: *SAINT*; program(s) used to solve structure: *SHELXTL* (Sheldrick, 2008 ▶); program(s) used to refine structure: *SHELXTL*; molecular graphics: *SHELXTL*; software used to prepare material for publication: *SHELXTL* and local programs.

## Supplementary Material

Crystal structure: contains datablock(s) I, global. DOI: 10.1107/S1600536812004564/qm2051sup1.cif

Structure factors: contains datablock(s) I. DOI: 10.1107/S1600536812004564/qm2051Isup2.hkl

Additional supplementary materials:  crystallographic information; 3D view; checkCIF report

## Figures and Tables

**Table 1:** Hydrogen-bond geometry (Å, °)

*D*—H⋯*A*	*D*—H	H⋯*A*	*D*⋯*A*	*D*—H⋯*A*
O6—H6*B*⋯O1^i^	0.85 (1)	2.06 (1)	2.887 (3)	167 (3)
O2—H2*B*⋯O6	0.71 (4)	1.98 (4)	2.681 (4)	172 (4)
O2—H2*A*⋯O5	0.81 (4)	2.63 (4)	3.078 (3)	116 (3)
O2—H2*A*⋯O3	0.81 (4)	1.85 (4)	2.652 (4)	170 (4)
O6—H6*A*⋯O4^ii^	0.84 (1)	2.02 (1)	2.831 (3)	162 (3)

Symmetry codes: (i) 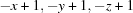; (ii) 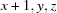.

**Fig. 1.**
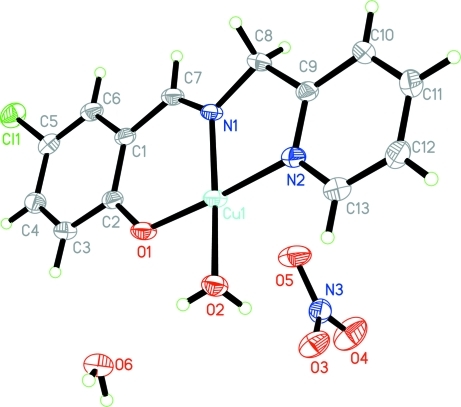
The molecular structure of (I), with atom labels and 30% probability displacement ellipsoids for non-H atoms.

**Fig. 2.**
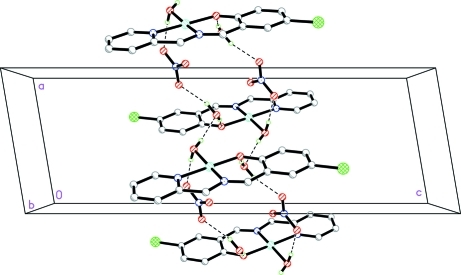
The packing of (I), viewed down the b axis. Hydrogen bonds are drawn as dashed lines. H atoms not involved in hydrogen bonding have been omitted.

## supplementary crystallographic information

### Comment

Schiff base copper(II) complexes have been received much attention due to their interesting structures and biological properties (Patel *et al.*, 2011; Creaven *et al.*, 2010; Osowole *et al.*, 2008). The title complex was first reported as triclinic space group P-1 (Liang *et al.*, 2010). We report herein a monoclinic polymorph in space group P21/c.

The title complex, (I) (Fig. 1), contains a mononuclear copper complex cation, a nitrate anion, and a water molecule. The Cu atom is coordinated by one phenolate O, one imine N, and one pyridine N of the Schiff base ligand, and one water O atom, forming a square planar coordination. The bond lengths (Table 1) are within the normal range. In the crystal, molecules are linked through intermolecular O—H···O hydrogen bonds (Table 2), to form chains along the *a* axis, Fig. 2.

### Experimental

To a solution of 5-chlorosalicylaldehyde (0.156 g, 1.0 mmol), 2-aminomethylpyridine (0.108 g, 1.0 mmol) in 30 ml me thanol was added slowly a solution of copper nitrate (0.241 g, 1.0 mmol) in methanol. The mixture was stirred for 2 h at room temperature to give a blue solution, which was filtered and the filtrate was left to stand at room temperature. Blue block crystals suitable for X-ray diffraction were obtained by slow evaporation.

### Refinement

The water H atoms were located in a difference map and refined with distances restraint of O—H = 0.85 (1) Å and H···H = 1.37 (2) Å. Other H atoms were positioned geometrically and refined using a riding model, with C—H = 0.93–0.97 Å.

### Crystal data

**Table:**

[Cu(C_13_H_10_ClN_2_O)(H_2_O)]NO_3_·H_2_O	*F*(000) = 828
*M**_r_* = 407.26	*D*_x_ = 1.720 Mg m^−^^3^
Monoclinic, *P*2_1_/*c*	Mo *K*α radiation, λ = 0.71073 Å
*a* = 7.840 (2) Å	Cell parameters from 3984 reflections
*b* = 8.815 (3) Å	θ = 2.5–26.9°
*c* = 23.079 (3) Å	µ = 1.60 mm^−^^1^
β = 99.680 (2)°	*T* = 298 K
*V* = 1572.4 (7) Å^3^	Block, blue
*Z* = 4	0.22 × 0.20 × 0.19 mm

### Data collection

**Table:**

Bruker SMART 1K CCD area-detector diffractometer	3410 independent reflections
Radiation source: fine-focus sealed tube	2647 reflections with *I* > 2σ(*I*)
Graphite monochromator	*R*_int_ = 0.046
ω scans	θ_max_ = 27.0°, θ_min_ = 2.5°
Absorption correction: multi-scan (*SADABS*; Sheldrick, 2004)	*h* = −9→9
*T*_min_ = 0.720, *T*_max_ = 0.752	*k* = −11→10
12290 measured reflections	*l* = −29→29

### Refinement

**Table:**

Refinement on *F*^2^	Primary atom site location: structure-invariant direct methods
Least-squares matrix: full	Secondary atom site location: difference Fourier map
*R*[*F*^2^ > 2σ(*F*^2^)] = 0.037	Hydrogen site location: inferred from neighbouring sites
*wR*(*F*^2^) = 0.089	H atoms treated by a mixture of independent and constrained refinement
*S* = 1.06	*w* = 1/[σ^2^(*F*_o_^2^) + (0.0362*P*)^2^ + 0.669*P*] where *P* = (*F*_o_^2^ + 2*F*_c_^2^)/3
3410 reflections	(Δ/σ)_max_ < 0.001
233 parameters	Δρ_max_ = 0.39 e Å^−^^3^
3 restraints	Δρ_min_ = −0.48 e Å^−^^3^

### Special details

**Table:**

Geometry. All e.s.d.'s (except the e.s.d. in the dihedral angle between two l.s. planes) are estimated using the full covariance matrix. The cell e.s.d.'s are taken into account individually in the estimation of e.s.d.'s in distances, angles and torsion angles; correlations between e.s.d.'s in cell parameters are only used when they are defined by crystal symmetry. An approximate (isotropic) treatment of cell e.s.d.'s is used for estimating e.s.d.'s involving l.s. planes.
Refinement. Refinement of *F*^2^ against ALL reflections. The weighted *R*-factor *wR* and goodness of fit *S* are based on *F*^2^, conventional *R*-factors *R* are based on *F*, with *F* set to zero for negative *F*^2^. The threshold expression of *F*^2^ > σ(*F*^2^) is used only for calculating *R*-factors(gt) *etc*. and is not relevant to the choice of reflections for refinement. *R*-factors based on *F*^2^ are statistically about twice as large as those based on *F*, and *R*- factors based on ALL data will be even larger.

### Fractional atomic coordinates and isotropic or equivalent isotropic displacement parameters (Å^2^)

**Table:**

	*x*	*y*	*z*	*U*_iso_*/*U*_eq_	
Cu1	0.31780 (4)	0.86046 (4)	0.441029 (14)	0.03273 (12)	
Cl1	0.30943 (12)	1.01565 (10)	0.74737 (3)	0.0561 (2)	
N1	0.2244 (3)	1.0428 (2)	0.46999 (9)	0.0311 (5)	
N2	0.2433 (3)	0.9578 (2)	0.36353 (9)	0.0341 (5)	
N3	0.0449 (3)	0.5644 (3)	0.39668 (10)	0.0427 (6)	
O1	0.3889 (3)	0.7778 (2)	0.51693 (8)	0.0387 (5)	
O2	0.4383 (4)	0.6939 (3)	0.40783 (11)	0.0392 (5)	
O3	0.1648 (3)	0.5200 (3)	0.37200 (10)	0.0539 (6)	
O4	−0.0834 (3)	0.4839 (3)	0.39510 (12)	0.0764 (8)	
O5	0.0556 (3)	0.6892 (3)	0.42285 (11)	0.0595 (6)	
O6	0.6908 (3)	0.5408 (3)	0.47630 (10)	0.0499 (5)	
C1	0.2878 (3)	0.9799 (3)	0.57295 (12)	0.0315 (6)	
C2	0.3686 (3)	0.8378 (3)	0.56748 (11)	0.0317 (6)	
C3	0.4316 (4)	0.7585 (3)	0.61943 (12)	0.0388 (7)	
H3	0.4867	0.6658	0.6170	0.047*	
C4	0.4142 (4)	0.8134 (3)	0.67321 (12)	0.0397 (7)	
H4	0.4573	0.7583	0.7069	0.048*	
C5	0.3323 (4)	0.9515 (3)	0.67803 (12)	0.0383 (7)	
C6	0.2709 (4)	1.0331 (3)	0.62901 (12)	0.0380 (7)	
H6	0.2169	1.1257	0.6327	0.046*	
C7	0.2211 (3)	1.0744 (3)	0.52380 (12)	0.0339 (6)	
H7	0.1715	1.1663	0.5317	0.041*	
C8	0.1576 (4)	1.1552 (3)	0.42524 (12)	0.0383 (7)	
H8A	0.2252	1.2476	0.4318	0.046*	
H8B	0.0385	1.1793	0.4279	0.046*	
C9	0.1674 (3)	1.0935 (3)	0.36544 (12)	0.0323 (6)	
C10	0.1044 (4)	1.1749 (3)	0.31527 (13)	0.0443 (7)	
H10	0.0529	1.2693	0.3177	0.053*	
C11	0.1198 (4)	1.1132 (4)	0.26167 (13)	0.0486 (8)	
H11	0.0773	1.1651	0.2272	0.058*	
C12	0.1979 (4)	0.9748 (4)	0.25938 (13)	0.0470 (8)	
H12	0.2096	0.9321	0.2234	0.056*	
C13	0.2586 (4)	0.9002 (3)	0.31071 (13)	0.0420 (7)	
H13	0.3122	0.8066	0.3090	0.050*	
H6A	0.773 (3)	0.535 (3)	0.4574 (12)	0.045 (10)*	
H2A	0.362 (5)	0.633 (4)	0.3958 (16)	0.067 (14)*	
H2B	0.502 (5)	0.656 (4)	0.4284 (15)	0.047 (12)*	
H6B	0.657 (5)	0.452 (2)	0.4820 (18)	0.100 (16)*	

### Atomic displacement parameters (Å^2^)

**Table:**

	*U*^11^	*U*^22^	*U*^33^	*U*^12^	*U*^13^	*U*^23^
Cu1	0.0389 (2)	0.02223 (18)	0.03751 (19)	0.00418 (15)	0.00787 (14)	−0.00095 (14)
Cl1	0.0715 (6)	0.0575 (5)	0.0400 (4)	−0.0038 (4)	0.0120 (4)	−0.0117 (4)
N1	0.0343 (12)	0.0188 (11)	0.0396 (12)	0.0034 (9)	0.0045 (10)	0.0015 (9)
N2	0.0384 (13)	0.0257 (12)	0.0381 (12)	−0.0011 (10)	0.0063 (10)	0.0001 (10)
N3	0.0444 (16)	0.0418 (16)	0.0400 (13)	−0.0038 (13)	0.0014 (11)	0.0011 (12)
O1	0.0533 (12)	0.0250 (10)	0.0385 (10)	0.0094 (9)	0.0100 (9)	−0.0021 (8)
O2	0.0399 (14)	0.0312 (12)	0.0468 (13)	0.0071 (11)	0.0079 (11)	−0.0014 (11)
O3	0.0568 (14)	0.0522 (14)	0.0567 (13)	−0.0071 (11)	0.0214 (11)	−0.0186 (11)
O4	0.0560 (16)	0.082 (2)	0.095 (2)	−0.0297 (15)	0.0250 (14)	−0.0259 (16)
O5	0.0533 (14)	0.0371 (13)	0.0913 (17)	0.0026 (11)	0.0211 (13)	−0.0148 (13)
O6	0.0554 (15)	0.0354 (13)	0.0579 (14)	0.0075 (11)	0.0067 (12)	−0.0017 (11)
C1	0.0319 (15)	0.0227 (13)	0.0401 (14)	−0.0018 (11)	0.0063 (12)	−0.0035 (11)
C2	0.0319 (15)	0.0252 (14)	0.0382 (14)	−0.0022 (11)	0.0064 (11)	−0.0022 (11)
C3	0.0438 (17)	0.0255 (15)	0.0464 (16)	0.0020 (13)	0.0057 (13)	0.0011 (12)
C4	0.0437 (18)	0.0359 (16)	0.0385 (15)	−0.0040 (13)	0.0042 (13)	0.0042 (13)
C5	0.0402 (17)	0.0370 (17)	0.0390 (15)	−0.0085 (13)	0.0099 (12)	−0.0094 (13)
C6	0.0402 (17)	0.0297 (15)	0.0451 (16)	0.0001 (13)	0.0099 (13)	−0.0076 (13)
C7	0.0329 (15)	0.0230 (13)	0.0460 (16)	0.0026 (12)	0.0073 (12)	−0.0037 (12)
C8	0.0460 (17)	0.0240 (14)	0.0438 (15)	0.0078 (13)	0.0040 (13)	0.0021 (12)
C9	0.0283 (14)	0.0245 (13)	0.0429 (15)	−0.0029 (11)	0.0021 (12)	−0.0002 (12)
C10	0.0478 (19)	0.0321 (16)	0.0468 (17)	0.0032 (14)	−0.0100 (14)	0.0017 (13)
C11	0.056 (2)	0.0434 (19)	0.0400 (16)	−0.0029 (16)	−0.0088 (14)	0.0048 (14)
C12	0.053 (2)	0.0446 (19)	0.0403 (16)	−0.0076 (16)	−0.0013 (14)	−0.0066 (14)
C13	0.0471 (18)	0.0327 (16)	0.0459 (17)	0.0002 (13)	0.0074 (14)	−0.0042 (13)

### Geometric parameters (Å, º)

**Table:**

Cu1—O1	1.8925 (18)	C2—C3	1.404 (4)
Cu1—N1	1.932 (2)	C3—C4	1.360 (4)
Cu1—O2	1.970 (2)	C3—H3	0.9300
Cu1—N2	1.981 (2)	C4—C5	1.389 (4)
Cl1—C5	1.735 (3)	C4—H4	0.9300
N1—C7	1.277 (3)	C5—C6	1.359 (4)
N1—C8	1.463 (3)	C6—H6	0.9300
N2—C9	1.340 (3)	C7—H7	0.9300
N2—C13	1.345 (3)	C8—C9	1.497 (4)
N3—O4	1.227 (3)	C8—H8A	0.9700
N3—O3	1.241 (3)	C8—H8B	0.9700
N3—O5	1.251 (3)	C9—C10	1.381 (4)
O1—C2	1.314 (3)	C10—C11	1.375 (4)
O2—H2A	0.81 (4)	C10—H10	0.9300
O2—H2B	0.71 (4)	C11—C12	1.371 (4)
O6—H6A	0.839 (10)	C11—H11	0.9300
O6—H6B	0.845 (10)	C12—C13	1.368 (4)
C1—C6	1.403 (4)	C12—H12	0.9300
C1—C2	1.419 (3)	C13—H13	0.9300
C1—C7	1.434 (4)		
			
O1—Cu1—N1	94.00 (8)	C5—C4—H4	119.9
O1—Cu1—O2	89.27 (9)	C6—C5—C4	120.1 (3)
N1—Cu1—O2	171.71 (10)	C6—C5—Cl1	121.2 (2)
O1—Cu1—N2	176.94 (8)	C4—C5—Cl1	118.7 (2)
N1—Cu1—N2	83.13 (9)	C5—C6—C1	121.0 (3)
O2—Cu1—N2	93.43 (10)	C5—C6—H6	119.5
C7—N1—C8	118.4 (2)	C1—C6—H6	119.5
C7—N1—Cu1	126.06 (19)	N1—C7—C1	125.3 (2)
C8—N1—Cu1	115.50 (16)	N1—C7—H7	117.3
C9—N2—C13	118.3 (2)	C1—C7—H7	117.3
C9—N2—Cu1	114.98 (18)	N1—C8—C9	109.7 (2)
C13—N2—Cu1	126.67 (19)	N1—C8—H8A	109.7
O4—N3—O3	118.9 (3)	C9—C8—H8A	109.7
O4—N3—O5	120.7 (3)	N1—C8—H8B	109.7
O3—N3—O5	120.3 (3)	C9—C8—H8B	109.7
C2—O1—Cu1	127.32 (17)	H8A—C8—H8B	108.2
Cu1—O2—H2A	105 (3)	N2—C9—C10	122.3 (3)
Cu1—O2—H2B	115 (3)	N2—C9—C8	116.5 (2)
H2A—O2—H2B	108 (4)	C10—C9—C8	121.2 (2)
H6A—O6—H6B	109 (2)	C11—C10—C9	118.4 (3)
C6—C1—C2	119.3 (2)	C11—C10—H10	120.8
C6—C1—C7	117.2 (2)	C9—C10—H10	120.8
C2—C1—C7	123.5 (2)	C12—C11—C10	119.6 (3)
O1—C2—C3	118.7 (2)	C12—C11—H11	120.2
O1—C2—C1	123.8 (2)	C10—C11—H11	120.2
C3—C2—C1	117.5 (2)	C13—C12—C11	119.1 (3)
C4—C3—C2	121.8 (3)	C13—C12—H12	120.4
C4—C3—H3	119.1	C11—C12—H12	120.4
C2—C3—H3	119.1	N2—C13—C12	122.2 (3)
C3—C4—C5	120.2 (3)	N2—C13—H13	118.9
C3—C4—H4	119.9	C12—C13—H13	118.9

### Hydrogen-bond geometry (Å, º)

**Table:**

*D*—H···*A*	*D*—H	H···*A*	*D*···*A*	*D*—H···*A*
O6—H6*B*···O1^i^	0.85 (1)	2.06 (1)	2.887 (3)	167 (3)
O2—H2*B*···O6	0.71 (4)	1.98 (4)	2.681 (4)	172 (4)
O2—H2*A*···O5	0.81 (4)	2.63 (4)	3.078 (3)	116 (3)
O2—H2*A*···O3	0.81 (4)	1.85 (4)	2.652 (4)	170 (4)
O6—H6*A*···O4^ii^	0.84 (1)	2.02 (1)	2.831 (3)	162 (3)

Symmetry codes: (i) −*x*+1, −*y*+1, −*z*+1; (ii) *x*+1, *y*, *z*.
